# Artificial neural network in orthodontic therapeutic extractions

**DOI:** 10.6026/973206300222646

**Published:** 2026-04-30

**Authors:** M.S Rohith, Shashanka P Kumar, Anusha G Hegde, Ajith Geevee, Pooja Mastammanavar, Apoorva Jha

**Affiliations:** 1 Department of Orthodontics and Dentofacial Orthopaedics, Vydehi Institute of Dental Sciences and Research Institute, Bangalore, India; 2 Department of Orthodontics and Dentofacial Orthopaedics, Vokkaligara Sangha Dental College and Hospital, Bengaluru, India; 3 Department of Orthodontics and Dentofacial Orthopaedics, Sri Dharmasthala Manjunatheshwara Dental College and Hospital, A constituent unit of Sri Dharmasthala Manjunatheshwara University, Dharwad, India; 4 Department of Orthodontics and Dentofacial Orthopaedics, Vinayaka Mission's Sankarachariyar Dental College, A constituent college of Vinayaka Mission's Research Foundation (Deemed to be University), Salem, India; 5 Department of Orthodontics and Dentofacial Orthopaedics, AJ Institute of Dental Sciences, Mangalore, India; 6 Department of Orthodontics and Dentofacial Orthopaedics, BGS Global Institute of Dental Sciences, Bangalore, India

**Keywords:** Artificial intelligence, artificial neural network, tracings, extraction

## Abstract

An unsubstantiated decision could result in several problems in its course as the extraction of teeth is an irreversible process. Therefore, it is of interest to develop an artificial intelligence decision making model for the diagnosis of extractions using neural network machine learning. The sample included 455 patients wherein input data consisted of 12 cephalometric variables and two additional indexes obtained from patients' records which were manually traced and digitized using NemoCeph 2D version 10software. It was observed that the accuracy of the binary classifier model, i.e., the decision of whether to extract or not, was 92.38 % and that of the multi- classifier model, i.e., the decision of which tooth to extract was also 92.38 %. Thus, we show that the technique of predicting orthodontic extractions using an artificial neural network is a reliable and valuable method.

## Background:

Determining the treatment plan is an integral part of orthodontic treatment. Deciding about extractions and the teeth to be extracted are essential parts of treatment planning because extractions are irreversible. Therefore, a judicial decision about extractions is to be made [1, 2-3]. During orthodontic treatment, an unsubstantiated decision could result in several problems in its course [4]. Undesirable results could be obtained, or the treatment might not be completed in the worst case [5]. Decisions with data by several orthodontists from the clinical evaluations, photographs, dental models and radiographs are done based on their own experience and knowledge [6, 7]. As treatment plans have no fixed formulae, the practitioner's learning method and experience leads to the decision in many cases. Intra-clinician and inter-clinician variation is often caused by this in the treatment planning process [8, 9]. The differences in treatment planning can occur between experienced and less-experienced clinicians [10]. Differences pertaining to extraction decisions could be critical [4]. While allowing inexperienced clinicians to learn from experienced practitioners would also be helpful, the standardization of decisions cannot be done with these combinations of measurements. Therefore, another approach is needed [11]. Many studies have been carried out about artificial intelligence and also in the field of bioinformatics. One of the approaches is machine learning using a neural network system. Machine learning fares better than human learning in situations that cannot be standardized [1]. The human neural system is composed of neurons linked at the synapse to send information. Each synapse linkage can be reinforced or weakened by repeated learning. In machine learning with a neural network, input to the output is linked by neurons and each neuron is connected at the synapse. In each synapse, the weighting technique collects information of the input neurons. Weighted values are adjusted through iterative learning. The goodness of fit of the training set can be elevated by excessive iterative learning. Nevertheless, the test set mistakes can also be increased; which is called overfitting. Introduction of a validation set is done to stop learning and making a generalized model would avoid this. The generalized decision-making model can be formed through these procedures [12]. Therefore, it is of interest to show an artificial intelligence decision- making model for diagnosing extractions using neural network machine learning and evaluate the model's validity and accuracy.

## Materials and Methodology:

## Inclusion criteria:

Persons included in the treatment plan groups-Non-extraction, Maxillary and mandibular first premolar extractions, Maxillary and mandibular second premolar extractions, only maxillary first premolar extraction, mandibular second premolar extraction and maxillary first premolar extractions.

## Exclusion criteria:

Patients with missing teeth (except 3rd molars), unerupted permanent teeth, malformed teeth, previous orthodontic treatment, maxillofacial deformities, orthognathic surgery were not included in the study. The place of study was V S Dental College and Hospital, Bengaluru. Two orthodontic specialists determined the treatment plans with more than ten years of experience. Four hundred and fifty-five lateral cephalograms were collected and traced as orthodontic records. A single investigator made all tracings. The reference points were digitized with the NemoCeph 2D version 10 software (Figure 1 - see PDF).

Twenty-six landmarks and fourteen measurements were chosen. The measurements in the cephalometry were:

[1] ANB angle is formed by joining Point A, Nasion and Point B.

[2] Overjet- Horizontal distance from the most proclined upper incisor to the labial surface of the lower incisor.

[3] Bjork sum- Sum of the angles formed by SN plane to S-Articulare, S-Articulare to Articulare Gonion and Articulare-Gonion to Mandibular plane.

[4] Overbite- Vertical overlap of the lower incisors by the upper incisors.

[5] Maxillary central incisor forms maxillary central incisor to SN angle to the Sella-Nasion plane.

[6] Maxillary central incisor to occlusal plane angle is formed by the long axis of the upper incisor to the occlusal plane.

[7] IMPA- Angle formed by the long axis of the lower incisor and the mandibular plane.

[8] Mandibular central incisor to occlusal plane angle is formed by the long axis of the lower incisor to the occlusal plane.

[9] Inter-incisal angle- Angle formed by the long axes of the upper and lower incisor.

[10] Upper lip to E-line- Horizontal distance of the most prominent point of the upper lip to the E lines (the line joining the tip of the nose and the chin).

[11] Lower lip to E-line- Horizontal distance of the most prominent point of the lower lip to the E line(which is the line joining the tip of the nose and the chin).

[12] The Nasolabial angle is formed by the line passing through the lower border of the nose and the upper lip. Apart from these, two other criteria were taken from the patients' record 

[13] Chief complaint score (0 indicated that the patient had forwardly placed teeth as the chief complaint and 1 indicated that the patient had other chief complaints).

[14] Arch length discrepancy.

The arch length discrepancy was measured in the upper and lower study models by bending a brass wire along with the cusp tips of the teeth (starting from the mesial surface of the first molar on one side to the other side), straightening the same and measuring the length and adding the individual mesiodistal width of teeth (second premolar on one side to the other side). The difference was noted. The last two criteria were obtained from the patients' records. Each patient was coded. A cellulose acetate sheet of 50 microns thickness was taped over each radiograph. The required anatomical landmarks were marked. Tracings were made on the sheet with a 0.3mm lead pencil, a millimetre-scale and a protractor (Figure 1 - see PDF). The above measurements were carried out after tracing. Photographs of the patient and models were provided to an orthodontist who had the experience of more than ten years to validate the treatment plan. 350 persons were assigned to the learning set from this sample and 105 persons were designated to the test set. The test set was used only for the evaluation of the models. Two-thirds of the learning set was assigned to the training set and one-third to the validation set. To find the optimal model, sliding window validation was performed. This is the validation technique to choose a validation set through the window moving sideways from the serial data. To prevent over-fitting, iterative learning was stopped at the minimum error point of the validation set. Next, the adequacy and accuracy were assessed by evaluating the test set and the best fit model was chosen. For training, TensorFlow 2.7 software was used with deep learning. Multiple layer neural network with ReLU activation and softmax on output for multiclass classification were used which resulted in poorer accuracy, possibly also due to limited input data leading to overfitting. For this, values with a known treatment plan were used to train the model. The number of samples was extended to 2660 by adding or subtracting 1 degree to Bjork sum and IMPA, which does not significantly alter the treatment plan. This was done to increase the model's accuracy as the accuracy in artificial intelligence depends on the sample size. Out of them, 2555 samples were used for training the model. After preparing the model, 105 values from the validation set were entered to predict the treatment plan.

[1] ANB angle is formed by joining Point A, Nasion and Point B.

[2] Overjet- Horizontal distance from the most proclined upper incisor to the labial surface of the lower incisor.

[3] Bjork sum- Sum of the angles formed by SN plane to S-Articulare, S-Articulare to Articulare Gonion and Articulare-Gonion to Mandibular plane.

[4] Overbite- Vertical overlaps of the lower incisors by the upper incisors.

[5] Maxillary central incisor forms maxillary central incisor to SN angle to the Sella-Nasion plane.

[6] Maxillary central incisor to occlusal plane angle is formed by the long axis of the upper incisor to the occlusal plane.

[7] IMPA- Angle formed by the long axis of the lower incisor and the mandibular plane.

[8] Mandibular central incisor to occlusal plane angle is formed by the long axis of the lower incisor to the occlusal plane.

[9] Inter-incisal angle- Angle formed by the long axes of the upper and lower incisor.

[10] Upper lip to E-line- Horizontal distance of the most prominent point of the upper lip to the E lines (the line joining the tip of the nose and the chin).

[11] Lower lip to E-line- Horizontal distance of the most prominent point of the lower lip to the E line(which is the line joining the tip of the nose and the chin).

[12] The Nasolabial angle is formed by the line passing through the lower border of the nose and the upper lip.

## Results:

This study consisted of lateral cephalograms of four hundred and fifty-five subjects. The study was conducted to develop an artificial intelligence decision-making model for diagnosing extractions using neural network machine learning and to determine the validity and accuracy of this model. Twenty-six landmarks were chosen on the lateral cephalograms and manually traced on the tracing sheet. Using NemoCeph 2D version 10 software (Figure 1 - see PDF), the same twenty-six landmarks were digitized. Twelve criteria were measured and recorded using these landmarks. The patient's chief complaint and arch length discrepancy were also considered from the patients' records. These were later used to train the artificial intelligence software. The first neural network determines whether a patient needs tooth extraction. If the patient needs extraction, one more neural network program predicts the specific extraction pattern, which can be all first premolar extraction (upper and lower), upper first premolar and lower second premolar extraction, all second premolar extraction (upper and lower), or upper first premolar extraction (Figure 2 - see PDF). A set of data comprising twelve criteria formed the training set, which is used to train the software where it correlates the data and creates a pattern of learning. When the test set of values was entered in the machine learning model, it predicted the chances of extraction or non-extraction (binary classifier system) with an accuracy of 92.38%. Within the extraction group, the accuracy for the teeth to be extracted (multi-classifier system) was 64.5 %. Later, after training with the extended sample size which is mentioned in the discussion, the accuracy rose to 92.38% (Figure 3 - see PDF).

## Discussion:

The decision to extract teeth for orthodontic treatment is critical because it is based on the clinician's experiences. Any aberration in the decision could lead to complications during orthodontic treatment. Undesirable results may be obtained, or the treatment might not even be completed in the worst-case scenario [1]. The process of diagnosis and treatment planning in orthodontics involves the identification of malocclusion and dentofacial abnormalities; establishing the etiology of the malocclusion; perceiving that treatment is possible; setting the treatment goals, designing a treatment plan to achieve the set goals. The decision to extract is based on the measurements of certain cephalometric analysis [4, 11 and 12]. But experienced orthodontists usually decide the same depending on their wisdom and knowledge. Extracting teeth as part of orthodontic treatment is governed mainly by concerns about facial appearance [5]. There can be differences regarding extraction or non-extraction between the various clinicians. Thus, a standard approach is required. One of the approaches that have attracted worldwide interest is the decision-making system of Artificial Neural networks [2]. This branch of engineering deals with computers' ability to understand and capability to imitate the functions of human brain to display intelligent behaviour and perform tasks with ease [13]. Machine learning is a subset of AI and was described as a program that learns to perform a task or automatically makes a decision from the data instead of having the behaviour explicitly programmed [14]. It is characterized by mathematical and statistical techniques enabling machines to improve their abilities by experience [15]. The idea of neural networks was inspired by the architecture of the neurons in the human brains [16, 17]. A simple neuron-A will receive an input from other neurons; when each of it is activated, it will cast a weighted "vote" for or against whether the neuron-A should itself activate [18]. An algorithm is needed for learning to adjust these weights depending on the training data; a simple algorithm (dubbed "fire together, wire together") would amplify the weight between two connected neurons when the activation of one neuron triggers the successful activation of another neuron [2]. Figures 4 and 5 (see PDF) illustrate the working of the machine learning model. There are certain random instances picked up from the training set and fed to the model which runs the test using the test data and the prediction is saved [16]. It also tests using the training data, the wrong predictions of which are updated back to the training set where they will be added as misclassified instances. Now, a random sample will be picked from these mixed samples of instances and misclassified instances and this process will continue for several times till the misclassified instances are negligible. The final prediction is obtained from the aggregate of the predictions. The most vital parts of orthodontic treatment would determine the treatment plan, outcomes and monitoring of patients. Deep learning uses several layers of neurons between the inputs and outputs of the network [19]. This study was different from the previous ones in with respect to the increased sample size and the absence of the patients' photographs. Higher-level features from the raw input can be extracted from the multiple layers. For example, lower layers in image processing may identify edges, whereas higher layers may identify the concepts relevant to a human, such as digits, letters, or faces [18]. This study uses deep learning for training the model. TensorFlow 2.7 software was used with deep learning for the Artificial Intelligence model. For the learning set, 355 five samples were incorporated and for the validation set, 105 samples were used. The cephalometric values of 355 tracings were utilized for deep learning. The number of samples for the training set was extended by adding and subtracting one degree to Bjork sum and IMPA, which would not affect the outcome decision or the accuracy. This was carried out to increase the sample size, which would amplify the model's accuracy. The extended sample size was 2660, out of which 2555 samples were used for training and validation and 105 were used for the test set. Photographs and models were provided to an orthodontic expert with more than ten years of experience to compare the model's accuracy. Similar to the study conducted by Seok-Ki Jung and Tae-Woo Kim, which had a sensitivity of 93% and a specificity of 84%, when a new set of values were entered into the model, it was able to predict the chances of extraction or non- extraction (binary classifier system) with an accuracy of 92.38%. Within the extraction group, the accuracy for the teeth to be extracted (multi-classifier system) was about 92.38%. The accuracy for predicting all second premolar extraction cases were low and the reason could be mainly due to the reduced number of samples available. The all first premolar extraction accuracy was the highest and the all second premolar extraction accuracy was the least. Interincisal angle and incisor mandibular plane angle (IMPA) affected the accuracy the most. The ANB angle and overbite were found to affect the same, least as they were already correlated with the previous criteria. Study concerning the use of artificial intelligence to be as accurate as human examiners, there should be at least 2300 sets of learning data, according to Moon *et al.* [19]. The reduced accuracy in this study could be due to the lesser number of samples. A larger sample size would have amplified the accuracy [20, 21 and 22]. Further study and research are required with an increased sample size for increased model accuracy. This study can be taken as the sole source to oversee the orthodontic treatment planning in patients and more parameters are to be considered in the upcoming studies. Different clinicians made the extraction decisions and the sample size was less pertinent to an artificial intelligence study.

## Conclusion:

t was found that the decision-making model was accurate enough to predict whether extraction is required for orthodontic treatment and the tooth to be extracted. The interincisal and Incisor mandibular plane angles affected the sensitivity and specificity the most and the ANB angle and overbite affected the same, least as they did not change the results. The accuracy of all first premolar extractions was the highest, whereas all second premolar extractions were the least.

## Funding:

This research did not receive any specific grant from funding agencies in the public, commercial or not-for-profit sector.
